# Matrix-Guided Size Selection of Plasmonic Nanoprobes for Improving Quantitative Robustness of SERS Lateral Flow Immunoassays

**DOI:** 10.3390/foods15183221

**Published:** 2026-09-11

**Authors:** Haowei Liu, Hao Wang, Shuo Zhang, Guoqiang Li, Xi Yu, Shengqi Yan, Limin Cao

**Affiliations:** 1 School of Medicine and Pharmacy, Ocean University of China, Qingdao 266005, China; 22130041012@stu.ouc.edu.cn; 2 State Key Laboratory of Marine Food Processing & Safety Control, College of Food Science and Engineering, Ocean University of China, 1299 Sansha Rd., Qingdao 266100, China

**Keywords:** SERS-LFIA, aflatoxin B_1_, gold nanoparticles, probe size, food safety

## Abstract

Surface-enhanced Raman scattering-based lateral flow immunoassays (SERS-LFIAs) have emerged as a promising platform for rapid food safety analysis. However, their quantitative reliability is often compromised by matrix-dependent variations during lateral flow, and the influence of probe size on analytical performance remains insufficiently understood. In this work, Au@DTNB immunoprobes with diameters of 15, 30, and 50 nm were prepared to systematically investigate the effect of nanoparticle size on the quantitative performance of SERS-LFIA for aflatoxin B1 (AFB1) detection. All three probe sizes exhibited excellent concentration-dependent responses and generated four-parameter logistic calibration curves with correlation coefficients (R2) of 0.997, 0.994, and 0.992, respectively. Although the 50 nm probes produced the strongest Raman signals, recovery experiments in rice and soybean samples revealed that the 15 nm probes provided the highest quantitative accuracy, with recoveries of 90.1–109.2% and 100.4–108.9%, respectively, and relative standard deviations below 7.2%. In contrast, larger probes exhibited progressively greater deviations from the spiked concentrations despite their stronger SERS responses. These results demonstrate that maximizing Raman signal intensity alone does not necessarily improve quantitative performance in complex food matrices. Instead, probe size should be optimized according to matrix characteristics to achieve reliable quantitative analysis. This work establishes a matrix-oriented strategy for probe size selection and provides practical guidance for the rational design of quantitative SERS-LFIA platforms for food safety analysis.

## 1. Introduction

Lateral flow immunoassays (LFIAs) are among the most widely adopted point-of-care analytical technologies [1] due to their low cost, portability, rapid response, and operational simplicity. These advantages have enabled broad applications in food safety monitoring, environmental analysis, and clinical diagnostics [2,3]. Nevertheless, conventional LFIAs predominantly rely on colorimetric readouts based on colloidal gold nanoparticles [4,5], which inherently limit analytical sensitivity and quantitative accuracy. In particular, colorimetric signals often exhibit poor resolution at low analyte concentrations and are susceptible to background interference and subjective interpretation [6]. Consequently, improving both sensitivity and quantitative reliability remains a central challenge in LFIA development [7]. Surface-enhanced Raman scattering (SERS) has been widely explored as a signal transduction strategy to overcome these limitations [8,9,10]. Owing to its ultrahigh sensitivity, molecular fingerprint specificity, and compatibility with lateral flow formats, SERS-based LFIAs have demonstrated substantially improved detection performance for diverse analytes [11]. However, despite these advantages, achieving robust quantitative analysis remains challenging in practical applications, where signal fluctuations and environmental perturbations frequently compromise measurement reliability [9].

To address these limitations, extensive efforts have been devoted to engineering SERS nanotags with enhanced signal intensity and improved signal stability [12,13,14,15,16]. Various plasmonic architectures, including core–shell nanoparticles, nanostars, nanoclusters, and hollow nanostructures [17,18,19,20], have been developed to increase electromagnetic field enhancement and improve Raman signal output. While these strategies have significantly improved signal strength at the nanoparticle level, quantitative performance in SERS-LFIA is not governed solely by intrinsic signal properties. As a dynamic chromatographic system, SERS-LFIA involves continuous nanoparticle transport through porous nitrocellulose membranes, followed by immunorecognition and accumulation at the test line [10,21]. Therefore, the final quantitative signal is determined not only by the Raman response of individual probes but also by the number of probes successfully transported and captured within the detection zone.

Since nanoparticle transport and capture within SERS-LFIA occur under continuous flow through porous membranes, these processes are highly sensitive to the surrounding physicochemical environment [16]. In practical applications, variations in sample matrices can significantly influence fluid transport behavior, nanoparticle mobility, and membrane–particle interactions, thereby affecting probe accumulation and signal formation at the test line [22]. Because these transport and capture processes are inherently size-dependent [23], their response to matrix variations is expected to differ for probes of different sizes, indicating that probe size selection may critically influence quantitative reliability across different sample matrices. However, a systematic understanding of how matrix-dependent transport behavior governs probe size selection in SERS-LFIA remains lacking.

**Scheme 1:**
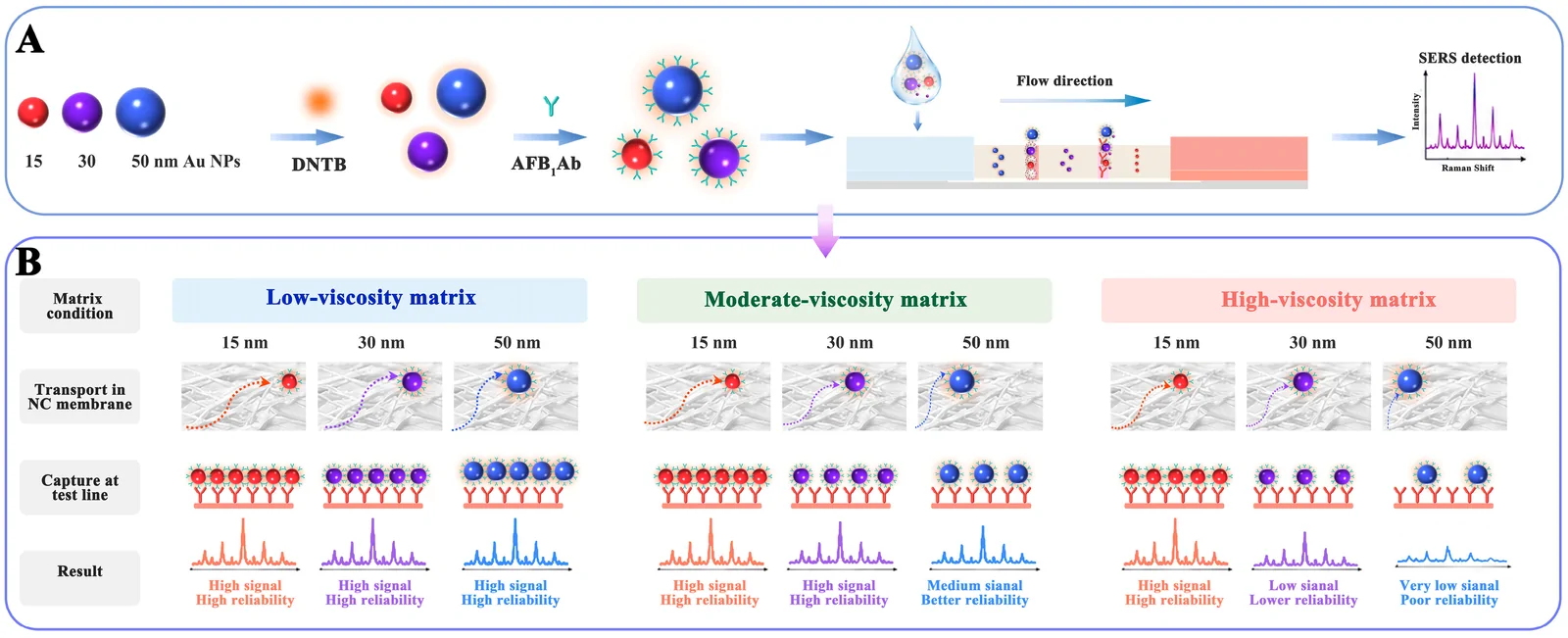
Structural design and probe-number-dependent quantitative SERS-LFIA strategy of Au@PNC-K_4_[Fe(CN)_6_] probes: (**A**) Preparation of SERS immunoprobes and principle of SERS lateral-flow immunoassay. (**B**) Transport, capture and SERS signal performance of 15 nm, 30 nm, 50 nm probes under different-viscosity matrices.

In this work, we systematically investigate the influence of sample matrix properties on the size-dependent quantitative performance of SERS-LFIA using three representative nanoprobe sizes (15, 30, and 50 nm) (Scheme 1). By correlating nanoparticle transport behavior, test-line accumulation, signal reproducibility, and quantitative analytical performance across matrices with distinct physicochemical properties, we elucidate the matrix-dependent nature of probe size effects in SERS-LFIA. Furthermore, we establish a mechanistic framework linking matrix properties, nanoparticle transport, and signal formation, thereby providing a rational basis for matrix-guided probe size selection to improve the quantitative reliability and robustness of SERS-based lateral flow immunoassays.

## 2. Materials and Methods

### 2.1. Reagents and Apparatus

Gold (III) chloride tetrahydrate (HAuCl_4_), sodium carbonate, sodium bicarbonate, trisodium citrate dihydrate, hydroxylamine hydrochloride, 5,5′-dithiobis (2-nitrobenzoic acid) (DNTB), and bovine serum albumin (BSA) were purchased from Sinopharm Chemical Reagent Co., Ltd. (Shanghai, China). Sample pads, nitrocellulose (NC) membranes, absorbent pads, and probe storage buffer were obtained from Nankairixin Biotechnology Co., Ltd. (Hangzhou, China). Aflatoxin B_1_ (AFB_1_), goat anti-mouse antibody (goat anti- mouse IgG), and AFB_1_-BSA conjugates were acquired from Sigma-Aldrich (Merck KGaA, Darmstadt, Germany). Whereas anti-AFB_1_ antibody (AFB_1_Ab) was self-prepared in our laboratory.

Raman spectra were acquired with an RMS1000 Raman spectrometer (Oceanhood, Shanghai, China). Ultraviolet-visible (UV-Vis) absorbance measurements were recorded using a Synergy LX multimode microplate reader (BioTek, Winooski, VT, USA). Transmission electron microscopy (TEM) characterizations were undertaken on a TECNAI G2 F20 field-emission transmission electron microscope (FEI, Hillsboro, OR, USA). Hitachi SU8010 field-emission scanning electron microscope (Hitachi, Tokyo, Japan) was utilized to obtain scanning electron microscopy (SEM) micrographs.

### 2.2. Synthesis of Size-Controlled Au Nps

**Synthesis of 15 nm AuNPs**

15 nm AuNPs were synthesized using the classical Frens citrate reduction method. A mixture of 98.9 mL of ultrapure water and 1 mL of 3 wt% trisodium citrate dihydrate solution was prepared in a dry conical flask and stirred at 400 rpm. The mixture was brought to boiling and maintained for 7 min. Subsequently, 0.1 mL of 10 wt% chloroauric acid trihydrate solution was rapidly injected, and the stirring speed was increased to 500 rpm. Boiling was maintained for an additional 7 min until the solution color stabilized, yielding monodisperse colloidal AuNPs with an average diameter of 15 nm.

**Synthesis of 30 nm AuNPs**

30 nm AuNPs were prepared via hydroxylamine-mediated seeded growth using the as-prepared 15 nm Au seeds. Specifically, 10 mL of the 15 nm Au seed solution was diluted with 9.4 mL of ultrapure water under stirring at 700 rpm. Freshly prepared 10 mM hydroxylamine hydrochloride solution (200 μL) and 1 wt% trisodium citrate dihydrate solution (200 μL) were added sequentially, followed by stirring for 5 min. Then, 400 μL of 1 wt% chloroauric acid trihydrate solution was introduced, and the reaction was allowed to proceed under continuous stirring at room temperature for 1 h to complete the seeded growth of the AuNPs.

**Synthesis of 50 nm AuNPs**

50 nm AuNPs were synthesized by seeded growth from the 15 nm Au seeds. Briefly, 1 mL of the 15 nm Au seed dispersion was diluted with 37.75 mL of ultrapure water and stirred at 700 rpm. Subsequently, 0.4 mL of 100 mM hydroxylamine hydrochloride solution and 0.4 mL of 1 wt% trisodium citrate dihydrate solution were added sequentially, followed by stirring for 5 min. A total of 450 μL of 1 wt% chloroauric acid trihydrate solution was then added dropwise in 50 μL aliquots. The reaction was maintained under continuous stirring at room temperature for 1 h to obtain 45 nm AuNPs. All AuNP colloidal suspensions were stored at 4 °C in the dark until further use.

### 2.3. Preparation of Dtnb-Labeled SERS Immunoprobes

Three sizes of AuNPs (15, 30, and 50 nm) were functionalized with DTNB to construct SERS nanotags and subsequently conjugated with antibodies to prepare SERS immunoprobes for lateral flow immunoassay. Briefly, each AuNP suspension was mixed with 0.1 mM DTNB solution at a volume ratio of 100:5. The mixture was stirred at 200 rpm for 10 min and then incubated for 1 h to obtain DTNB-labeled Au nanoparticles (Au@DTNB NPs). The resulting Au@DTNB NP suspension was then mixed with 0.1 M sodium carbonate–sodium bicarbonate buffer (pH 9.16) at a volume ratio of 15:1 and equilibrated for 1 min. AFB_1_Ab was subsequently added under continuous stirring at 200 rpm to a final concentration of 2 μg/mL, followed by incubation for 25 min to complete antibody conjugation. To block nonspecific binding sites, 10% (m/v) BSA solution was added to the probe suspension at a volume ratio of 20:1, and the mixture was incubated for 20 min. The resulting immunoprobes were collected by centrifugation at different speeds according to particle size (8000 rpm for 15 nm AuNPs, 6000 rpm for 30 nm AuNPs, and 4000 rpm for 50 nm AuNPs). Finally, the precipitated SERS immunoprobes were resuspended in probe storage buffer to one-quarter of the original volume for subsequent lateral flow immunoassay.

### 2.4. Preparation of SERS-LFIA Strips

The fabrication of SERS-LFIA strips was initiated by mounting an NC membrane onto a plastic backing card. AFB_1_-BSA conjugate and goat anti-mouse IgG were then dispensed onto the NC membrane as the test line (T line) and control line (C line) at concentrations of 1 mg/mL and 0.5 mg/mL, respectively. The membrane was dried at 37 °C for 2.5 h to immobilize the proteins. Subsequently, the sample pad and absorbent pad were attached to the backing card with 2–3 mm overlaps onto the NC membrane to ensure continuous capillary flow. Finally, the assembled sheets were cut into individual strips with a width of 3 mm using a strip cutter. The prepared SERS-LFIA strips were stored in a dry and dark environment until use.

### 2.5. Lateral Flow Immunoassay Procedure and SERS Measurement

For SERS-LFIA detection, 100 μL of sample solution (buffer, starch suspension, or soybean extract), 100 μL of SERS immunoprobe suspension, and 100 μL of probe storage buffer were mixed thoroughly and incubated for 5 min at room temperature. The SERS-LFIA strip was then vertically immersed into the mixture, allowing the solution to migrate along the strip via capillary action and enabling competitive immunoreactions to occur on the test and control lines. After complete chromatographic migration (approximately 5 min), the strip was removed and air-dried at room temperature prior to SERS measurement.

SERS signals were collected directly from the test line using an RMS1000 Raman spectrometer equipped with a 785 nm excitation laser. All Raman spectra were acquired under identical instrumental conditions. The characteristic Raman peak of DTNB at 1330–1332 cm^−1^ was selected for quantitative analysis, and all spectra were baseline-corrected before signal extraction. Quantitative analysis was performed by correlating the average Raman intensity at 1330 cm^−1^ with the concentration of AFB_1._ Unless otherwise specified, each experiment was independently performed in triplicate.

### 2.6. Recovery Experiments in Real Samples

Rice and soybean samples were purchased from a local supermarket in Qingdao, China. The samples were individually ground into fine powders using a homogenizer prior to analysis. Blank samples were spiked with AFB_1_ at final concentrations of 0.5, 1, and 2 ng/g, followed by thorough mixing to ensure homogeneous distribution of the analyte.

For sample preparation, the spiked powders were mixed with lateral flow running buffer diluted two-fold with ultrapure water at a solid-to-liquid ratio of 0.5 g/mL. The mixtures were vortexed thoroughly and centrifuged at 4000 rpm for 5 min. Subsequently, 200 μL of the supernatant was mixed with 100 μL of the SERS immunoprobe suspension and incubated for 5 min. The SERS-LFIA strip was then vertically immersed into the mixture for competitive detection. After complete chromatographic migration, SERS measurements were performed as described in Section 2.5. The AFB_1_ concentrations were calculated using the corresponding four-parameter logistic calibration equations established for each probe size, and the recoveries were determined based on the measured and spiked concentrations.

## 3. Results and Discussion

### 3.1. Construction and Characterization of Au@dtnb SERS Nanotags with Different Sizes

**Figure 1:**
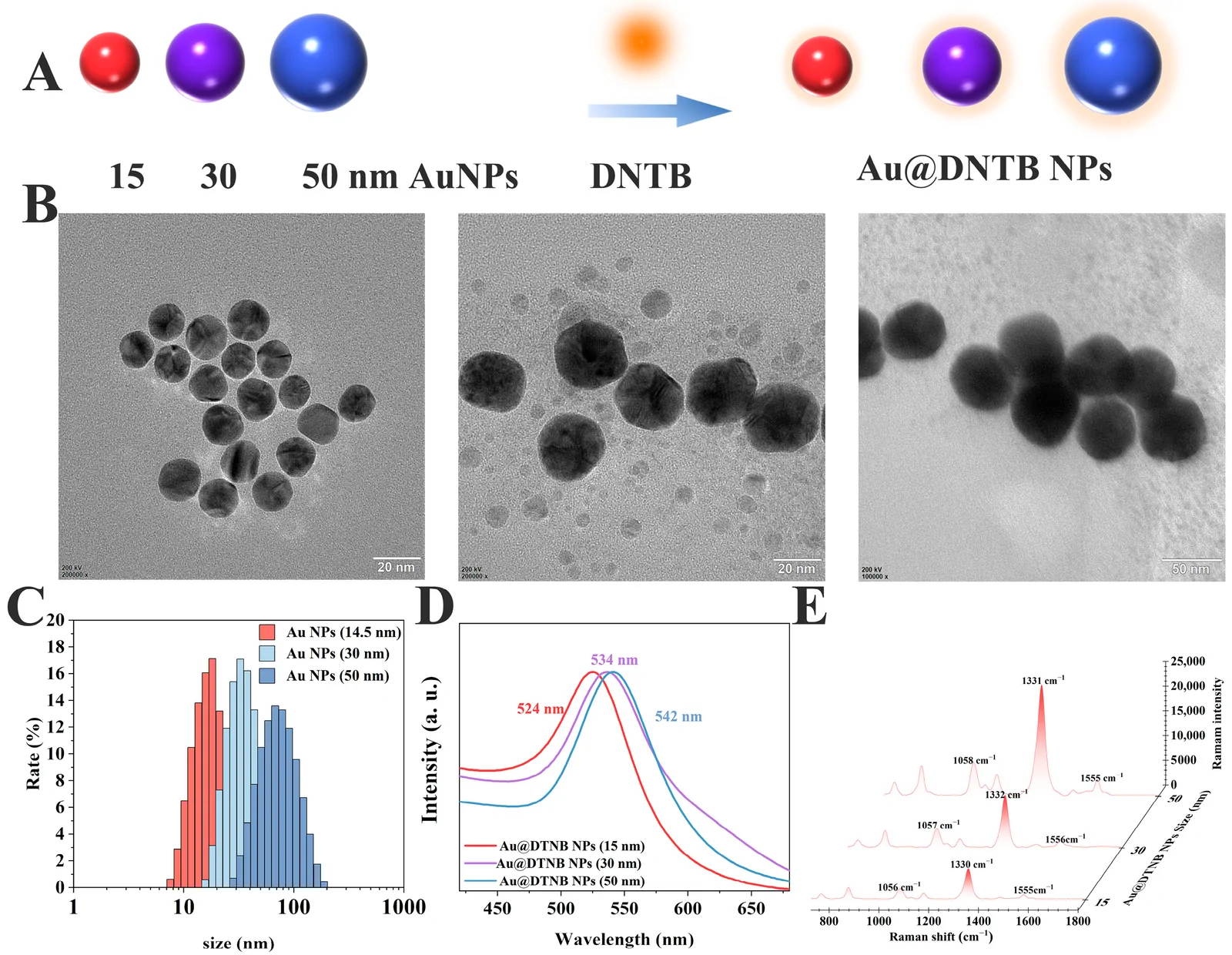
Preparation and characterization of Au@DTNB SERS nanotags with different particle sizes. (**A**) Schematic illustration of the preparation of Au@DTNB nanotags using Au nanoparticles with core diameters of 15, 30, and 50 nm. (**B**) TEM images of the corresponding Au@DTNB nanotags. (**C**) Particle size distributions determined from TEM images. (**D**) UV–vis absorption spectra of Au@DTNB nanotags with different particle sizes. (**E**) SERS spectra of Au@DTNB nanotags acquired under identical experimental conditions.

To establish SERS nanotags with distinct plasmonic properties for subsequent SERS-LFIA investigations, spherical Au nanoparticles with average diameters of 15, 30, and 50 nm were synthesized and subsequently modified with DTNB, as illustrated in Figure 1A. DTNB was employed as the Raman reporter through Au–S interactions to construct size-dependent Au@DTNB nanotags while maintaining identical surface chemistry among the three nanoparticle systems.

The morphology and structural integrity of the prepared Au@DTNB nanotags were first examined by transmission electron microscopy. As shown in Figure 1B, all nanoparticles exhibited regular spherical morphologies with smooth surfaces and good monodispersity after DTNB modification. No apparent aggregation or structural deformation was observed, indicating that the adsorption of DTNB did not affect the colloidal stability of the nanoparticles. Statistical analysis of the TEM images (Figure 1C) further confirmed narrow particle size distributions with average diameters centered at approximately 15, 30, and 50 nm, respectively, demonstrating excellent size controllability and reproducibility of the synthetic strategy.

The optical properties of the three Au@DTNB nanotags were characterized by UV-vis spectroscopy. As presented in Figure 1D, the localized surface plasmon resonance (LSPR) peak gradually shifted from 524 nm for the 15 nm nanotags to 534 nm and 542 nm for the 30 and 50 nm counterparts, respectively. This progressive red shift agrees well with the size-dependent plasmonic behavior of spherical Au nanoparticles, confirming the successful preparation of Au@DTNB nanotags with distinct optical characteristics.

The Raman spectra of Au@DTNB nanotags with different particle sizes are presented in Figure 1E. All three nanotags exhibited identical characteristic Raman fingerprints of DTNB, confirming the successful immobilization of DTNB molecules onto the Au nanoparticle surface through Au-S interactions. The characteristic bands observed at approximately 1056–1058 cm^−1^, 1330–1332 cm^−1^, and 1555–1556 cm^−1^ can be assigned to the C-H in-plane bending vibration of the aromatic ring, the symmetric stretching vibration of the nitro (-NO_2_) group, and the C=C stretching vibration of the aromatic ring, respectively. Among these characteristic bands, the Raman band centered at approximately 1331 cm^−1^ exhibited the highest intensity and excellent spectral resolution and was therefore selected as the analytical signal for subsequent quantitative SERS-LFIA measurements. In addition, the Raman intensity increased progressively with increasing nanoparticle size, indicating enhanced electromagnetic field amplification associated with larger plasmonic nanoparticles. These results demonstrate that increasing nanoparticle size effectively improves the intrinsic SERS activity of individual nanotags. However, the analytical performance of SERS-LFIA is determined not only by the intrinsic Raman enhancement of the probes but also by their transport and capture behavior during lateral flow. Therefore, these well-defined Au@DTNB nanotags provide an appropriate model system for systematically investigating the influence of probe size on quantitative SERS-LFIA performance under different sample matrix conditions.

### 3.2. Characterization of Au@dtnb Immunoprobes with Different Sizes

To construct SERS immunoprobes for lateral flow immunoassays, anti-AFB_1_ monoclonal antibodies were immobilized onto the Au@DTNB nanotags through electrostatic adsorption. The physicochemical properties of the resulting immunoprobes were systematically characterized to verify successful surface functionalization and evaluate their colloidal stability.

**Figure 2:**
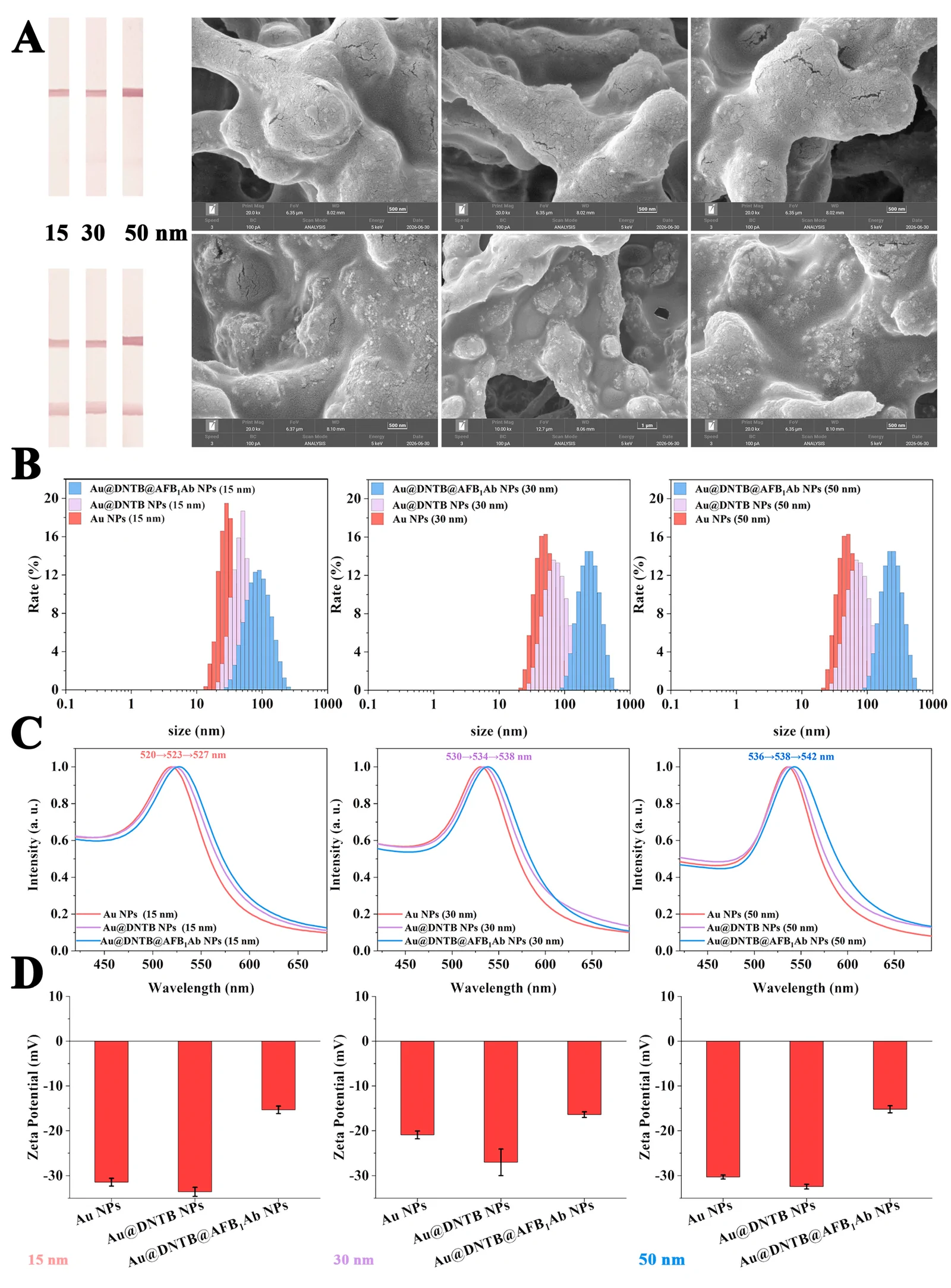
Physicochemical characterization of Au@DTNB immunoprobes with different particle sizes. (**A**) Representative photographs of SERS-LFIA strips developed using Au@DTNB immunoprobes (**left**) and SEM images of the corresponding test lines after lateral flow (**right**). (**B**) Hydrodynamic size distributions of Au nanoparticles, Au@DTNB nanotags, and Au@DTNB immunoprobes measured by DLS. (**C**) UV–vis absorption spectra of Au nanoparticles, Au@DTNB nanotags, and Au@DTNB immunoprobes during stepwise surface functionalization. (**D**) Zeta potentials of Au nanoparticles, Au@DTNB nanotags, and Au@DTNB immunoprobes.

Representative photographs of the assembled SERS-LFIA strips and scanning electron microscopy (SEM) images of the corresponding test lines are presented in Figure 2A. Distinct test and control lines were observed for all three probe sizes, indicating that antibody conjugation did not impair the chromatographic migration or immunorecognition capability of the probes. SEM images further revealed dense accumulation of nanoparticles within the porous nitrocellulose membrane after lateral flow, confirming the efficient capture of the immunoprobes at the test line.

Dynamic light scattering (DLS) measurements (Figure 2B) revealed a gradual increase in hydrodynamic diameter following DTNB modification and subsequent antibody conjugation for each nanoparticle size. Compared with the original Au nanoparticles, both Au@DTNB nanotags and Au@DTNB immunoprobes exhibited larger hydrodynamic diameters, consistent with the sequential formation of DTNB and protein layers on the nanoparticle surface. Importantly, the particle size distributions remained relatively narrow throughout the modification process, indicating that probe preparation did not induce noticeable aggregation. The UV-vis absorption spectra further confirmed the successful surface modification (Figure 2C). For each nanoparticle size, slight red shifts in the LSPR absorption peak were observed after DTNB modification and antibody immobilization. Specifically, the absorption maxima shifted from 520 to 523 and 527 nm for the 15 nm nanoparticles, from 530 to 534 and 538 nm for the 30 nm nanoparticles, and from 536 to 538 and 542 nm for the 50 nm nanoparticles. These spectral changes can be attributed to the increase in the local refractive index surrounding the Au nanoparticles following molecular adsorption, while the absence of obvious peak broadening indicates that the probes maintained good colloidal dispersion during surface functionalization.

Changes in surface charge were further examined by zeta potential measurements. As shown in Figure 2D, DTNB modification rendered the nanoparticles more negatively charged because of the exposed nitro and carboxyl groups of DTNB. After antibody immobilization, the absolute zeta potential decreased for all three nanoparticle sizes, reflecting the successful adsorption of protein molecules onto the nanoparticle surface. Despite these changes, all immunoprobes remained sufficiently negatively charged to maintain colloidal stability.

Collectively, the DLS, UV-vis, and zeta potential analyses consistently verify the successful fabrication of Au@DTNB immunoprobes with different particle sizes while preserving favorable colloidal stability. Since the three immunoprobes exhibited comparable physicochemical characteristics after surface modification, the influence of probe preparation on subsequent analytical performance can be largely excluded, allowing the effect of nanoparticle size on SERS-LFIA performance to be systematically investigated.

### 3.3. Matrix-Dependent Signal Stability of SERS Immunoprobes with Different Sizes

To investigate the influence of sample matrices on the signal stability of SERS immunoprobes with different sizes, four representative blank matrices, including assay buffer, cellulose suspension, starch suspension, and extracted soybean protein solution, were evaluated using the same SERS-LFIA procedure. The use of blank matrices excluded the influence of competitive immunoreactions, allowing the matrix effect on probe migration and signal generation to be assessed independently.

**Figure 3:**
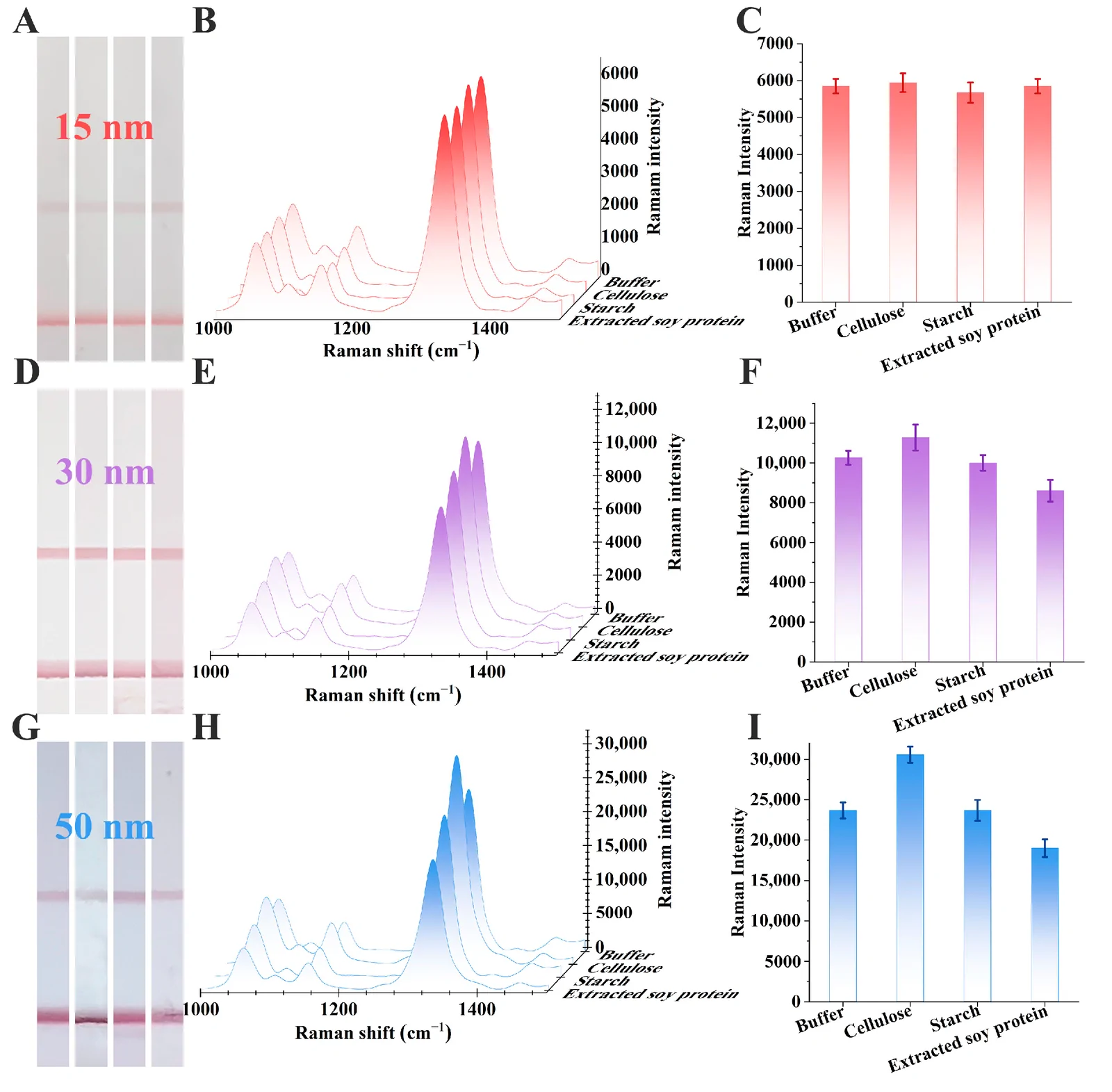
Representative SERS-LFIA strips obtained using (**A**) 15 nm, (**D**) 30 nm, and (**G**) 50 nm Au@DTNB immunoprobes under different blank matrices (buffer, cellulose, starch, and extracted soybean protein, from left to right). Corresponding SERS spectra are shown in (**B**,**E**,**H**), and the Raman intensities at 1330 cm^−1^ are summarized in (**C**,**F**,**I**). Data are presented as mean ± SD (*n* = 3).

As shown in Figure 3A, distinct test and control lines were obtained for all three probe sizes in assay buffer, cellulose, starch, and extracted soybean protein, indicating that all immunoprobes maintained satisfactory chromatographic migration and immunorecognition capability regardless of the matrix composition. Meanwhile, differences in the color intensity of the test lines were observed among the four matrices, and the extent of these variations increased with probe size. The 15 nm probes exhibited relatively consistent color development across all matrices, whereas more pronounced differences were observed for the 30 and 50 nm probes, suggesting that the colorimetric stability of SERS-LFIA is influenced by both probe size and sample matrix.

To further evaluate the influence of different matrices, Raman spectra were collected directly from the test lines. As shown in Figure 3B,E,H, the characteristic SERS spectra remained essentially unchanged under all matrix conditions, whereas the Raman intensity varied significantly with both matrix composition and probe size. Among these characteristic bands, the Raman peak at approximately 1330 cm^−1^, which exhibited the highest intensity and excellent spectral stability, was selected as the quantitative signal throughout this study. Although the spectral profiles remained essentially identical, noticeable differences in Raman intensity were observed among the four matrices, and the extent of these variations became increasingly dependent on probe size. The 15 nm probes exhibited nearly identical Raman intensities in all matrices, whereas progressively larger fluctuations were observed for the 30 nm and 50 nm probes.

The quantitative comparison of the Raman intensity at 1330 cm^−1^ is summarized in Figure 3C,F,I. For the 15 nm probes, the Raman intensities measured in buffer, cellulose, starch, and extracted soybean protein were highly consistent, indicating excellent signal stability regardless of matrix composition. In contrast, the 30 nm probes exhibited moderate matrix-dependent fluctuations, while the 50 nm probes displayed the largest signal variation. Notably, the Raman intensity reached its highest value in the cellulose matrix and decreased substantially in the extracted soybean protein matrix, with buffer and starch exhibiting intermediate signal levels. These results demonstrate that the magnitude of matrix-induced signal variation increases progressively with nanoparticle size, indicating that larger probes are more susceptible to changes in sample matrix composition.

Because identical Raman reporters, probe preparation procedures, and measurement conditions were employed throughout the experiments, the observed signal differences cannot be attributed to changes in the intrinsic Raman activity of the probes. Instead, they are more reasonably associated with differences in probe transport and accumulation during chromatographic migration. Larger nanoparticles are expected to experience stronger steric hindrance and greater interactions with matrix components within the porous nitrocellulose membrane, resulting in increased variability in the number of probes reaching the test line. Consequently, although larger probes provide higher intrinsic Raman intensity, their signal output becomes increasingly dependent on the surrounding matrix environment. These findings demonstrate that probe size is an important determinant of matrix-dependent signal stability in SERS-LFIA and provide the experimental basis for the subsequent evaluation of quantitative analytical performance under different sample matrix conditions.

### 3.4. Probe Size-Dependent Quantitative Response of SERS-LFIA

**Figure 4:**
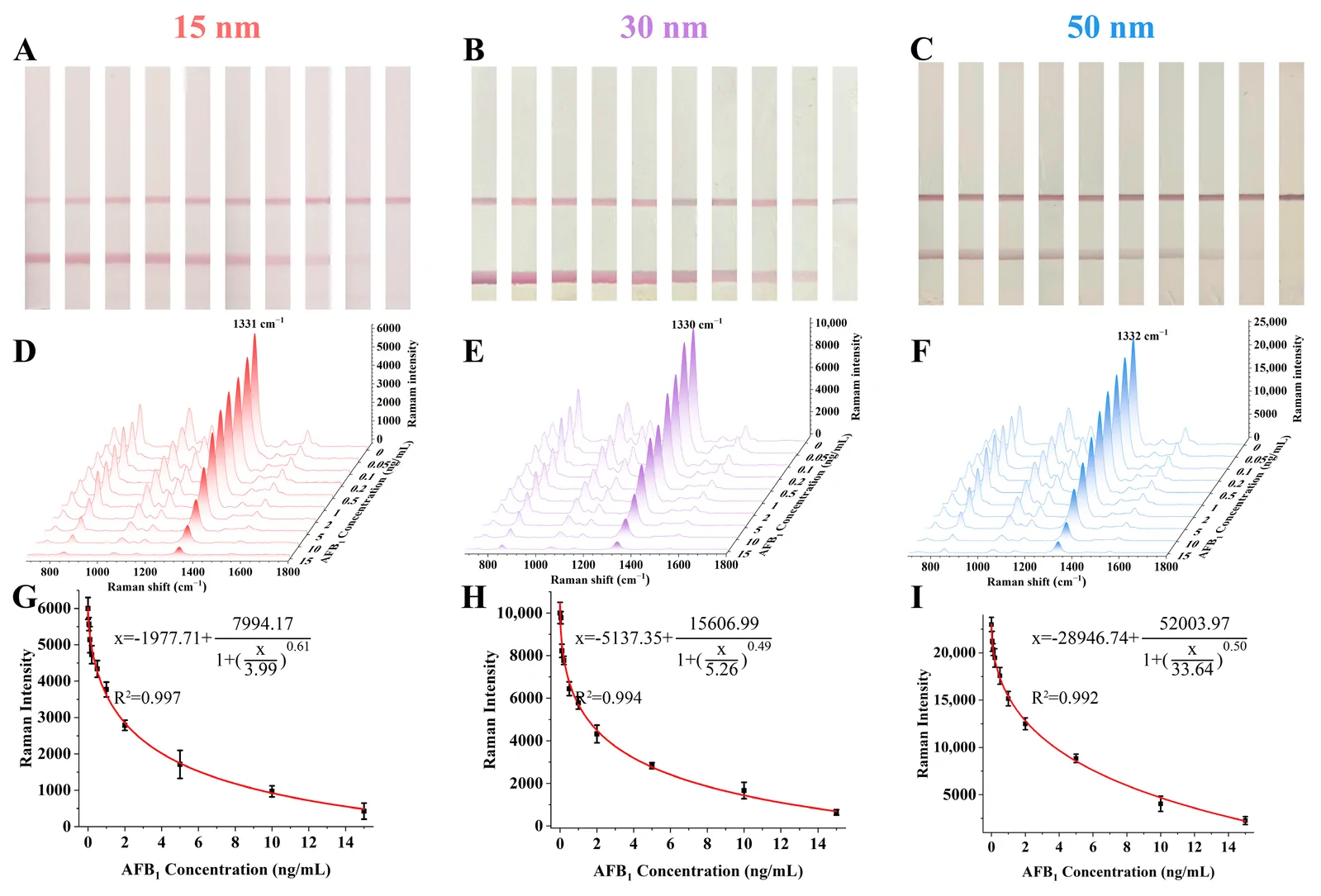
Representative SERS-LFIA strips (**A**–**C**), corresponding SERS spectra collected from the test lines (**D**–**F**), and calibration curves established from the Raman intensity at 1332 cm^−1^ (**G**–**I**) for AFB_1_ detection using 15, 30, and 50 nm Au@DTNB immunoprobes. Data are presented as mean ± SD (*n* = 3).

To evaluate the quantitative response of SERS-LFIA constructed with different-sized immunoprobes, competitive immunoassays were performed using 15, 30, and 50 nm Au@DTNB immunoprobes. Representative photographs of the test strips are shown in Figure 4A–C. As the concentration of AFB_1_ increased from 0 to 15 ng mL^−1^, the color intensity of the test line gradually decreased for all three probe sizes, while the control line remained clearly visible throughout the assay. These results demonstrate that all immunoprobes maintained satisfactory chromatographic migration and competitive immunorecognition during the lateral flow process. The detection of AFB_1_ was based on a competitive immunoassay, in which free AFB_1_ competes with AFB1-BSA immobilized at the test line for binding to the anti-AFB1 antibodies on the Au@DTNB probes. Thus, increasing AFB_1_ concentrations result in fewer probes captured at the test line and consequently weaker DTNB Raman signals.

The corresponding SERS spectra collected from the test lines are presented in Figure 4D–F. For all three probe sizes, the characteristic Raman peak of DTNB at approximately 1332 cm^−1^ gradually decreased with increasing AFB_1_ concentration, reflecting the progressive reduction in the number of immunoprobes captured on the test line as free AFB_1_ competitively inhibited probe binding. The consistent attenuation of the Raman signal confirms that all three immunoprobes effectively converted competitive immunorecognition into concentration-dependent SERS responses. Although the three probe sizes exhibited similar concentration-dependent response trends, their signal intensities differed considerably. Under identical assay conditions, the 15 nm immunoprobes generated the lowest Raman intensity throughout the entire concentration range, whereas the 50 nm immunoprobes produced the strongest SERS response, with the 30 nm probes exhibiting intermediate signal levels. This trend is consistent with the characterization results shown in Figure 1 and Figure 3, indicating that increasing nanoparticle size enhances the intrinsic Raman output of individual SERS immunoprobes.

The quantitative calibration curves derived from the Raman intensity at 1332 cm^−1^ are shown in Figure 4G–I. All three probe sizes exhibited typical sigmoidal concentration–response relationships and were well fitted using a four-parameter logistic model. The corresponding fitting equations are presented in Figure 4, yielding correlation coefficients (R^2^) of 0.997, 0.994, and 0.992 for the 15, 30, and 50 nm immunoprobes, respectively. The estimated LODs were 0.034, 0.047, and 0.017 ng/mL, while the corresponding LOQs were 0.250, 0.692, and 0.175 ng/mL for the 15, 30, and 50 nm probes, respectively, calculated from the residual standard deviations of the respective 4PL regressions as described in Supporting Information, Section S1. The excellent fitting performance demonstrates that SERS-LFIA constructed with different-sized immunoprobes enables reliable quantitative determination of AFB_1_ within the investigated concentration range. Compared with gold-standard HPLC and LC-MS/MS, which demand large instruments and tedious sample pretreatment, the present SERS-LFIA achieves AFB_1_ detection in 10 min with a portable Raman spectrometer (Table S1, Supporting Information). Notably, probe-size tuning reveals a trade-off between SERS sensitivity and matrix anti-interference performance (Section 3.3), providing practical guidance for grain sample testing.

### 3.5. Validation of Probe Size-Dependent Quantitative Performance in Real Samples

To further evaluate whether the calibration models established under standard conditions could accurately predict analyte concentrations in practical sample analysis, recovery experiments were performed using rice and soybean samples spiked with AFB_1_ at three concentration levels. The Raman intensities collected from the test lines were converted into AFB_1_ concentrations using the corresponding four-parameter logistic calibration equations established in Figure 4, and the recoveries were subsequently calculated from the measured and spiked concentrations.

The analytical results are summarized in Table 1. For rice samples, the 15 nm immunoprobes provided recoveries ranging from 90.1% to 109.2% with RSDs of 3.3–5.4%, indicating satisfactory analytical accuracy and precision. The recoveries obtained using the 30 nm probes ranged from 101.9% to 122.4%, while those obtained using the 50 nm probes further increased to 125.5–133.2%, despite comparable analytical precision. A similar trend was observed for soybean samples. The 15 nm immunoprobes yielded recoveries of 100.4–108.9%, whereas the recoveries obtained using the 30 and 50 nm probes increased to 107.1–122.4% and 135.7–149.8%, respectively. The corresponding RSD values remained below 7.2% for all measurements, demonstrating satisfactory repeatability.

Although all three probe sizes exhibited excellent calibration performance under standard conditions (Figure 4), obvious differences in recovery were observed in rice and soybean samples. The 15 nm immunoprobes consistently produced recoveries closest to the spiked values, whereas the recoveries gradually deviated with increasing probe size despite the stronger Raman signals generated by larger nanoparticles. This discrepancy indicates that probe size affects the susceptibility of the assay to matrix-induced deviations in practical samples. Compared with the buffer-based calibration, the complex sample extracts may alter probe dispersion, nonspecific interactions, chromatographic migration, and antibody–antigen competitive binding, thereby changing the relationship between Raman response and analyte concentration. Such effects become increasingly evident with increasing probe size, as reflected by the progressively higher recoveries obtained with the 30 and 50 nm immunoprobes in both rice and soybean samples. These results indicate that calibration models established under buffer conditions cannot be directly translated into accurate quantitative analysis in complex food matrices, where probe size significantly influences the consistency between the calibration model and the actual analytical response. Combined with the results presented in Figure 3 and Figure 4, these findings demonstrate that probe size should be optimized according to matrix conditions rather than Raman signal intensity alone to achieve reliable quantitative SERS-LFIA.

To further assess the analytical selectivity of the developed SERS-LFIA, additional cross-reactivity experiments were performed using structurally related aflatoxins (AFB_2_, AFG_1_, AFG_2_, and AFM_1_) and other common mycotoxins, including ochratoxin A (OTA), zearalenone (ZEA), T-2 toxin, and HT-2 toxin. AFB_1_ was tested at 1 ng/mL, whereas the other mycotoxins were evaluated at 10 ng/mL. The corresponding test-strip (30 nm) responses are provided in Figure S1 (Supporting Information). The results showed a clear response to AFB_1_, whereas the tested non-target mycotoxins produced substantially weaker responses under the selected conditions. The structurally related aflatoxins exhibited a certain degree of cross-reactivity, while the responses to ochratoxin A (OTA), zearalenone (ZEA), T-2 toxin, and HT-2 toxin were close to those of the blank. These results indicate that the assay response is primarily governed by antibody-mediated competitive recognition and that the observed cross-reactivity toward related aflatoxins is associated with their structural similarity to AFB_1_.

## 4. Conclusions

In this work, the influence of probe size on the quantitative performance of SERS-LFIA under different matrix conditions was systematically investigated using Au@DTNB immunoprobes with diameters of 15, 30, and 50 nm. Although increasing nanoparticle size significantly enhanced the intrinsic SERS signal intensity, stronger Raman signals did not necessarily translate into improved quantitative accuracy. All three probe sizes exhibited satisfactory calibration performance under standard conditions, whereas distinct differences were observed in practical sample analysis. Recovery experiments in rice and soybean samples demonstrated that the 15 nm immunoprobes provided the most accurate and reliable quantitative results, indicating superior adaptability to complex food matrices. These findings reveal that probe size is a critical factor governing the quantitative reliability of SERS-LFIA and should be optimized according to matrix conditions rather than Raman enhancement alone. This work provides a practical strategy for probe size selection and offers useful guidance for the rational design of reliable SERS-LFIA platforms for food safety analysis.

## Figures and Tables

**Table 1:** Recovery of AFB_1_ in real samples using SERS immunoprobes with different sizes (*n* = 3).

	Samples		Rice			Soybean		
Size		Added (ng/g)	Found (ng/g)	Recovery (%)	RSD	Found (ng/g)	Recovery (%)	RSD
15 nm		0.5	0.503	100.6	4.6	0.536	107.2	4.05
		1	0.901	90.1	5.4	1.004	100.4	3.45
		2	2.184	109.2	3.3	2.178	108.9	1.80
30 nm		0.5	0.612	122.4	5.7	0.657	131.4	7.20
		1	1.019	101.9	3.6	1.306	130.6	5.40
		2	2.204	110.2	4.2	2.143	107.15	4.65
50 nm		0.5	0.645	129	5.4	0.749	149.8	4.05
		1	1.332	133.2	4.5	1.357	135.7	4.95
		2	2.511	125.55	5.7	2.75	137.5	6.30

## Data Availability

The original contributions presented in this study are included in the article/Supplementary Material. Further inquiries can be directed to the corresponding author.

## Supplementary Materials

The following supporting information can be downloaded at: https://www.mdpi.com/article/10.3390/foods15183221/s1. Figure S1. Selectivity of the SERS-LFIA toward AFB1 and other mycotoxins. Table S1. Comparison of representative analytical methods for aflatoxin B1 determination in food matrices. References [24,25] are cited in the Supplementary Materials.
