# Supplementary material for: Matrix-Guided Size Selection of Plasmonic Nanoprobes for Improving Quantitative Robustness of SERS Lateral Flow Immunoassays
Source: Foods. 2026 Sep 11;15(18):3221. doi: 10.3390/foods15183221 (PMC13605682; doi:10.3390/foods15183221)
Supplement: Supplementary file 1 [file foods-15-03221-s001.zip › foods-4479632-supplementary.pdf]

Supporting information

Matrix-Guided Size Selection of Plasmonic Nanoprobes for Improving Quantitative Robustness of SERS Lateral Flow Immunoassays

Section S1. Calibration model and analytical performance

The quantitative relationship between AFB_1 concentration and Raman intensity for Au@DTNB SERS-LFIA probes (15 nm, 30 nm, 50 nm) was evaluated using a four-parameter logistic (4-PL) calibration model. A series of AFB_1 standard concentrations were used for calibration, and the SERS Raman intensity was plotted against AFB_1 concentration. The general 4-PL equation for a competitive immunoassay is expressed as follows:

$$\mathbf{y=A_2+(A_1-A_2)/(1+(x/x_0)^p)}$$

where y represents the SERS Raman intensity response; x represents the AFB_1 concentration (ng/mL); A_1 is the asymptotic signal response at zero AFB_1 concentration (blank upper plateau); A_2 is the asymptotic signal response at infinitely high AFB_1 concentration (inhibited lower plateau); x_0 is the inflection-point concentration; and p is the Hill slope factor.

Residual standard deviation (σ) derived from the residuals between experimental calibration points and 4-PL fitted curves was used to estimate the assay noise. Considering the inverse-response characteristic of competitive SERS-LFIA (SERS signal decreases as AFB_1 concentration increases), the threshold signal for LOD was calculated by offsetting the blank plateau response A_1 downward by 3.3σ:

$$\mathbf{y_{LOD} = A_1 - 3.3\sigma}$$

The threshold signal for LOQ was obtained by offsetting A_1 downward by 10σ:

$$\mathbf{y_{LOQ} = A_1 - 10\sigma}$$

Section S2. Selectivity evaluation

The selectivity of the proposed SERS-LFIA was evaluated by comparing the T-line Raman responses toward AFB_1 and other common mycotoxins. AFB_1 was tested at 1 ng/mL, while AFB_2, AFG_1, AFG_2, AFM_1, OTA, ZEA, T-2 toxin, and HT-2 toxin were tested at 10 ng/mL. The corresponding T-line signal reduction was calculated as follows:

$$R(\%)=\left(1-\frac{I_{sample}}{I_{blank}}\right)\times 100$$

These parameters were used to compare the competitive responses induced by the target and non-target mycotoxins.

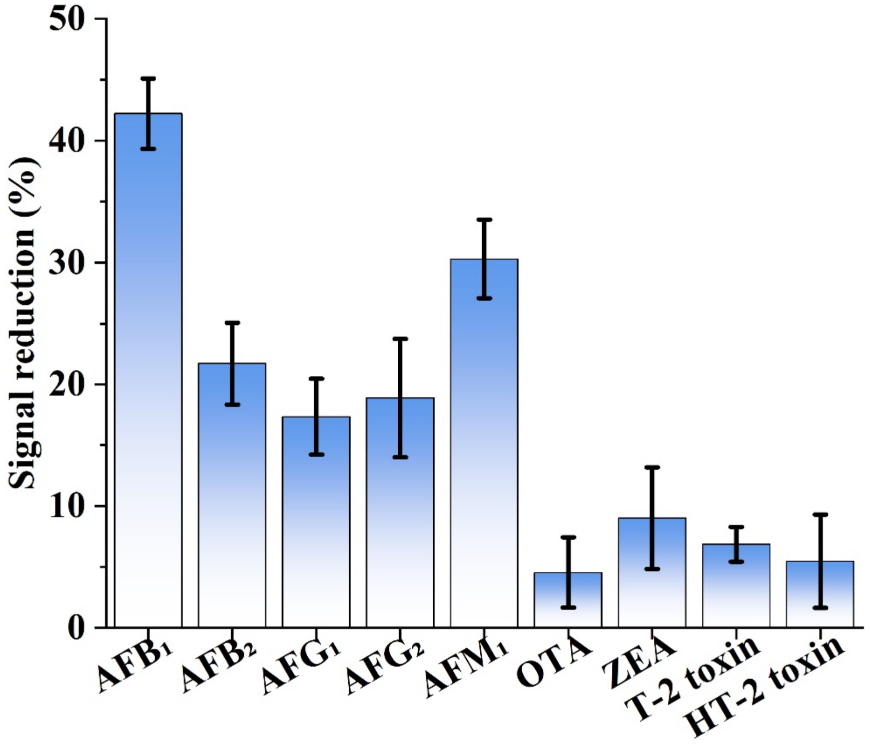

Figure S1. Selectivity of the SERS-LFIA toward AFB_1 and other mycotoxins.

Table S1. Comparison of representative analytical methods for aflatoxin B1 determination in food matrices.

Method	Target	Assay time	Matrix	Instrument	Main advantage
[1]	AFB1	—	—	HPLC	High analytical accuracy
[2]	AFB1, AFB2, AFG1, AFG2	—	—	LC-MS/MS	High sensitivity and confirmatory capability
[3]	AFB1, ZEN	20 min	Corn	Raman spectrometer	Rapid SERS-LFIA screening
[4]	AFB1, ZEN, DON	20 min	Corn	Portable Raman	On-site multi-mycotoxin screening
[5]	—	—	Serum	Handheld Raman	Improved reproducibility and stability
This work—15 nm	AFB1	10 min	Rice, soybean	SERS-LFIAs	Improved quantitative robustness
This work—30 nm	AFB1	10 min			Intermediate performance
This work—50 nm	AFB1	10 min			Highest intrinsic SERS intensity

[1] Ji, C.; Sun, X.; Fang, Y.; Li, P. Determination of Aflatoxin B1 in Grains by Aptamer Affinity Column Enrichment and Purification Coupled with High Performance Liquid Chromatography Detection. *Foods* **2024**, *13*, 640. DOI: 10.3390/foods13050640.

[2] Lijalem, Y. G.; Gab-Allah, M. A.; Yu, H.; Choi, K.; Kim, B. Development of isotope dilution-ultrahigh-performance liquid chromatography-tandem mass spectrometry for the accurate determination of aflatoxins in grains. *J. Food Compos. Anal.* **2024**, *126*, 105896. DOI: 10.1016/j.jfca.2023.105896.

[3] Yin, L.; Cai, J.; Ma, L.; You, T.; Arslan, M.; Jayan, H.; Zou, X.; Gong, Y. Dual function of magnetic nanocomposites-based SERS lateral flow strip for simultaneous detection of aflatoxin B1 and zearalenone. *Food Chem.* **2024**, *446*, 138817. DOI: 10.1016/j.foodchem.2024.138817.

[4] Yin, L.; Ma, L.; Jia, W.; Gong, Y.; Cai, J. Portable SERS-based lateral flow strip for simultaneous rapid detection of three typical corn mycotoxins. *Food Chem.* **2026**, *502*, 147677. DOI: 10.1016/j.foodchem.2025.147677.

[5] Sloan-Dennison, S.; Clark, B.; Scullion, K. M.; Smillie, F.; Laing, S.; Fineran, P.; Mair, J.; Rathmell, C.; Bingemann, D.; Faircloth, J.; Creasey, D.; Shand, N. C.; Weir, C. J.; Dear, J. W.; Graham, D.; Faulds, K. Development of a surface enhanced Raman scattering lateral flow immunoassay with prolonged reproducibility and stability over time. *Analyst* **2026**, *151*, 3130–3140. DOI: 10.1039/D6AN00053C.
